# Human Sialic acid *O*-acetyl esterase (SIAE) – mediated changes in sensitivity to etoposide in a medulloblastoma cell line

**DOI:** 10.1038/s41598-019-44950-5

**Published:** 2019-06-13

**Authors:** Rebecca L. Mather, Katie F. Loveson, Helen L. Fillmore

**Affiliations:** 1 University of Portsmouth, Brain Tumour Research Centre, School of Pharmacy and Biomedical Sciences, St. Michael’s Building, White Swan Road, Portsmouth, UK; 2 The Open University, School of Life, Health and Chemical Sciences, Walton Hall, Milton Keynes, Buckinghamshire UK

**Keywords:** Cancer, Cell biology

## Abstract

Medulloblastoma (MB), the most common malignant paediatric brain tumour occurs in the cerebellum. Advances in molecular genomics have led to the identification of defined subgroups which are associated with distinct clinical prognoses. Despite this classification, standard therapies for all subgroups often leave children with life-long neurological deficits. New therapeutic approaches are therefore urgently needed to reduce current treatment toxicity and increase survival for patients. GD3 is a well-studied ganglioside which is known to have roles in the development of the cerebellum. Post-partum GD3 is not highly expressed in the brain. In some cancers however GD3 is highly expressed. In MB cells GD3 is largely acetylated to GD3^A^. GD3 is pro-apoptotic but GD3^A^ can protect cells from apoptosis. Presence of these gangliosides has previously been shown to correlate with resistance to chemotherapy. Here we show that the GD3 acetylation pathway is dysregulated in MB and as a proof-of-principle we show that increased GD3 expression sensitises an MB cell line to etoposide.

## Introduction

Medulloblastoma (MB) is the most common malignant brain tumour of childhood and remains the leading cause of cancer-related mortality in children up to the age of fourteen^1^. MBs arise in the cerebellum and can spread to the spinal cord. Although recent studies have identified multiple subgroups, four distinct molecular subgroups have been defined and are now recognised by the World Health Organisation. The major subgroups consist of WNT, SHH, Group 3 and Group 4^2–5^.

Despite a reported survival rate of >5 years post-diagnosis of 80.1%, (60–70% for high-risk children)^3,6^, a proportion of patients remain incurable due to leptomeningeal dissemination^7,8^. Where MB survival can be achieved, it is often accompanied with a severe impact on the patient’s quality of life. This impact is due to treatment-induced life-long neurological deficits and endocrine disorders^9^. Novel therapeutic strategies are therefore urgently needed to improve survival for patients, and to improve quality of life for survivors.

Gangliosides are glycosphingolipids which contain at least one sialic acid residue in their oligosaccharide chain^10^. Gangliosides have been shown to have diverse roles in physiology, and pathology (including cancer)^11^. One of the most characterized gangliosides in the brain is GD3^12–14^. GD3 is a predominant ganglioside in neural stem cells, where it has been shown to play a role in proliferation via epidermal growth factor receptor signaling^15^. GD3 and its acetylated form GD3^A^ are both known to be expressed by granule neuron precursors (GPCs) during cerebellar development^16^, and these cells are implicated in the pathology of SHH MB^17^. In rats, GD3^A^ is particularly enriched at sites of axonal contact between cells of the external granule layer and molecular layer^18^. Post-partum GD3 accumulates within the supernumerary GPCs and causes mitochondria-mediated apoptosis^19–21^. Expression of GD3 in the post-partum brain is virtually absent^22,23^ however in some cancers of neural crest origin, such as malignant melanoma, and glioblastoma (GBM)^24–26^ these gangliosides are expressed. GD3^A^ has been has been shown to prevent GD3 from its apoptotic-associated role^27^ and in addition, has been implicated in leukemia to confer resistance to chemotherapeutic agents^28^.

GD3 and GD3^A^ can exist in a delicate balance between pro-apoptotic GD3 and pro-survival GD3^A^ ^24,29^. Two enzymes are responsible for the turnover of these gangliosides. The GD3 acetylation enzyme remains elusive, however there is some evidence that Cas 1 domain containing 1 (Casd1)^30,31^ is the enzyme responsible for this process. The GD3^A^ deacetylation enzyme is known to be sialic acid *O*-acetyl esterase (SIAE)^32,33^. As GD3^A^ is thought to protect cells from mitochondria-mediated apoptosis, we designed *proof-of-concept* experiments to determine if deacetylation of GD3^A^ by SIAE would result in increased GD3, and cause cells to become apoptotic or sensitise them to existing chemotherapies.

## Results

### Expression of CASD1 and SIAE varies according to molecular subgroup

**Figure 1:**
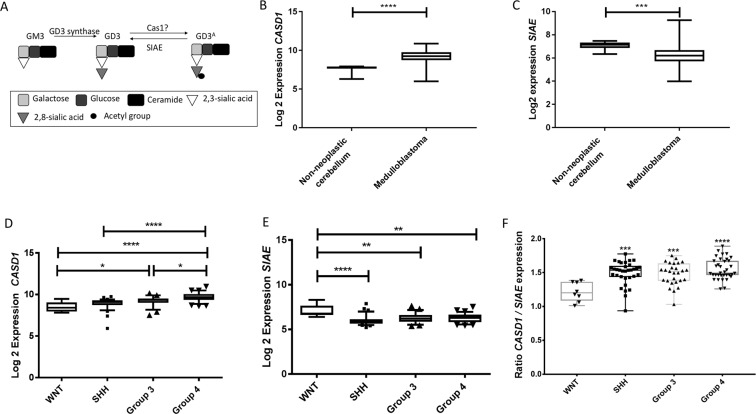
Expression of GD3 acetylation pathway components in clinical MB samples. (**A)** Schematic of the GD3 acetylation pathway shows the roles of GD3 synthase, SIAE and CASD1 in the synthesis and turnover of GD3 and GD3^A^. (**B**) Expression of *CASD1*, encoding the enzyme that converts GD3 to GD3^A^ is upregulated in MB samples (Pfister dataset) compared to non-neoplastic cerebellum (Roth dataset; p < 0.0001). (**C**) Expression of *SIAE*, encoding the enzyme which deacetylates GD3^A^ to GD3 is significantly down-regulated in MB samples (Pfister dataset) compared to non-neoplastic cerebellum (Roth dataset; p = 0.0005). (**D**) Expression of *CASD1* is highest in group 4 samples when compared to WNT and SHH (p < 0.0001), and group 3 (p = 0.0221). The expression of *CASD1* is also significantly higher in group 3 compared to WNT (p = 0.00403). (**E**) Expression of *SIAE*, is significantly higher in WNT tumours compared to SHH (p < 0.0001), group 3 (p = 0.00043), and group 4 (p = 0.0031). Data obtained from the R2 database using the Northcott data set (gse21140). (**F**) The data from **D** and **E** analysed to generate a ratio of expression between *CASD1* and *SIAE* for each sample. There is a significant inverse-correlation between *CASD1* and *SIAE* expression in SHH, group 3, and group 4 subgroups when compared to WNT (SHH p = 0.0003; Group 3 p = −0.0008; Group 4 p < 0.0001). Data was analysed by (**B** and **C**) two-tailed unpaired t-test; (**D–F**) one-way analysis of variance (ANOVA) followed by Tukey’s multiple comparisons post hoc test using graph pad prism 6 software. (^*^p < 0.05; ^**^p < 0.01; ^****^p < 0.0001).

We first sought to examine the expression of genes involved in the acetylation and deacetylation of GD3 in MB using publicly available data from the R2 database (r2.amc.nl); the details of individual datasets used can be found in materials and methods. The components of the GD3 pathway are summarised in Fig. 1A. Briefly, GD3 is synthesised by the enzyme GD3 synthase. GD3 can then be acetylated by the enzyme CASD1, and deacetylated by the enzyme SIAE.

We next analysed data from non-neoplastic cerebellar samples obtained at autopsy (Roth GSE3526)^34^ and compared expression with MB. Expression of CASD1 was significantly up-regulated in MB samples (Fig. 1B p < 0.0001). In contrast SIAE, GD3’s deacetylation enzyme was significantly down-regulated in MB samples (Fig. 1C p = 0.0005). Due to chip types we were unable to corroborate this data using other datasets. Next, to determine if CASD1 and SIAE were dysregulated according to subgroup we used the largest dataset available which has samples with annotated molecular subgroups (Northcott (GSE21140))^35^. CASD1 was found to be highest in group 4. Further to this, expression of CASD1 in groups 3 and 4 was significantly higher than WNT (p < 0.0001; Fig. 1D), significantly higher in group 4 compared to SHH (p < 0.0001; Fig. 1C), and expression was also higher in group 4 compared to group 3 (p = 0.0221). Finally, and in contrast, expression of SIAE was shown to be significantly lower in SHH (p < 0.0001), group 3 (p = 0.0042) and group 4 (p < 0.0031) compared to WNT patients (Fig. 1E). The data from Fig. 1D,E was then analysed to generate a ratio of expression between CASD1 and SIAE for each sample (Fig. 1F). This data demonstrates that there is a significant inverse-correlation between CASD1 and SIAE expression in SHH, group 3, and group 4 subgroups when compared to WNT (SHH p = 0.0003; Group 3 p = −0.0008; Group 4 p < 0.0001). This data was further corroborated using all publicly available datasets on the R2 database which contained samples annotated by molecular subgroup (Supplementary Fig. 1).

### GD3 and GD3^A^ are expressed in MB cell lines

**Figure 2:**
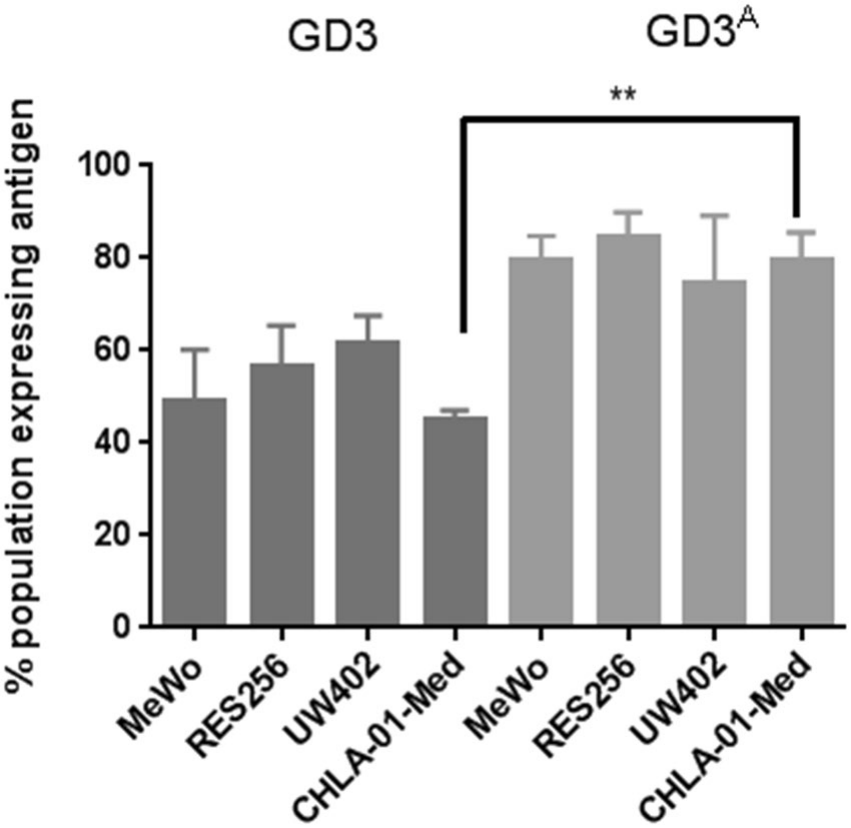
Expression of GD3 and GD3^A^ was confirmed by flow cytometry. Intracellular expression of GD3 and GD3^A^ demonstrates a significantly higher percentage of cells expressing GD3^A^ compared to GD3 in CHLA-01-Med (p = 0.0011). Data analysed by unpaired two-tailed t-test using Graph Pad Prism 6 software (^**^p < 0.01; ^***^p < 0.001) n = 3 ± SEM.

As we have determined the expression of GD3 pathway components in MB clinical samples was dysregulated, we sought to determine the function of this pathway using *in vitro* models of MB. Expression of GD3 and GD3^A^ in MB was then investigated using established cell lines. Flow cytometric analysis of GD3 and GD3^A^ showed that the population of cells expressing GD3 was similar for the three cell lines RES256, UW402 and CHLA-01-Med (56.7%, 61.3% and 45.1% respectively; Fig. 2). The percentage of the cells expressing GD3^A^ was also found to be similar between the cell lines (85.4%, 74.4% and 79.4% respectively; Fig. 2). Expression of GD3^A^ was slightly, but not significantly, higher than GD3 expression in RES256 and UW402, (85.4% and 74.4% respectively), however GD3^A^ expression was significantly higher in CHLA-01-Med (79.4%, Fig. 2 p = 0.0305).

### Induction of SIAE in RES256 cells

**Figure 3:**
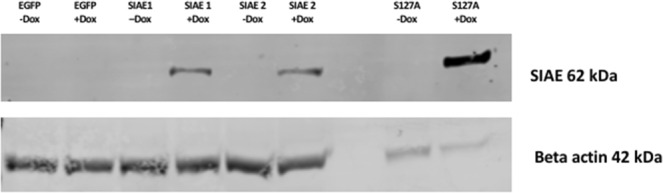
SIAE expression is induced by stable tet-responsive RES256 cells. RES256 cells were cultured in the presence (+) and absence (−) of doxycycline (dox) for 48 hours and analysed by western blot. SIAE was expressed by two wild-type clones, SIAE 1 and SIAE 2, and by the catalytic mutant, S127A. Importantly only when the cells were cultured with doxycycline for 48 hours was SIAE expressed. Equal protein loading between samples from each clone are confirmed with beta actin. A further immuno-reactive band of approximately 32 kDa for SIAE was seen (Supplementary Fig. 3). The identity of this band is unknown. Less protein was loaded in the S127A samples due to protein concentration of the lysate. Image acquired using the Licor Odyssey Clx equipped with ImageStudio 5 software. Representative image of n = 2.

SIAE is responsible for the deacetylation of 9-*O*-acetylated sialic acid residues such as those found in GD3^A^^32^. We hypothesised that deacetylation of GD3^A^ would increase GD3 levels and therefore induce mitochondria-mediated apoptosis. To investigate this premise, an inducible system was used to induce SIAE expression in the MB cell line RES256. SIAE expression was found low in RES256 cells without SIAE overexpression, which was expected based on the previous bioinformatic analyses. Following validation of expression, one EGFP vector control clone, two wild-type SIAE clones, and one previously described SIAE catalytically inactive mutant (S127A)^33^ clone were maintained for subsequent experiments. Transcription of genes were confirmed indirectly via expression of EGFP (Supplementary Fig. 2). SIAE expression was induced for 48 h with doxycycline and confirmed by western blot analysis (Fig. 3). We found SIAE expression to be undetectable at the protein level in RES256 cells without doxycycline addition, which was unsurprising based on bioinformatic analyses of MB tissue (Fig. 1 and Supplementary Fig. 1). SIAE is predicted to have a molecular weight of 62 kDa and this was confirmed. Western blot analysis also demonstrated no SIAE expression by EGFP clones when treated with doxycycline (Fig. 3). SIAE clones 1 and 2 express SIAE only in the presence of doxycycline. Interestingly, the catalytically mutated S127A clone, when treated with doxycycline, expressed more SIAE protein than the WT clones. The reason for this is currently unknown.

### SIAE expression increases esterase activity in RES256 cells

**Figure 4:**
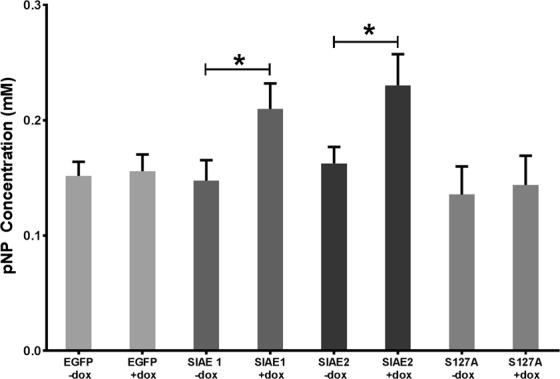
Esterase activity is increased with SIAE induction. Total esterase activity was investigated using an esterase substrate (pNP) assay. Expression of SIAE was induced in RES256 cells for 48 hours using doxycycline. RES256 cells were treated with 0.2 mM pNP in the presence and absence of doxycycline. Only SIAE expressing clones demonstrated a significant increase in esterase activity with induction of expression, SIAE 1 (p = 0.0447) and SIAE 2 (p = 0.0434). No significant increases in activity were seen with induction of EGFP or S127A clones (p > 0.05). Data analysed by one-way analysis of variance (ANOVA) and Siddak post-hoc test using GraphPad Prism 6 software n = 3.

To determine if the induced SIAE was functional in RES256 cells, esterase activity was investigated using the esterase substrate 4-nitrophenol (pNP). The vector control (EGFP) and the catalytic mutant (S127A) clone showed no increase in esterase activity following induction with doxycycline (p > 0.05; Fig. 4). Both wild-type SIAE clones 1 and 2 demonstrated a significantly increased esterase activity when treated with 0.2 mM pNP, only when cells were treated with doxycycline (SIAE 1 p = 0.0447; SIAE 2 p = 0.0434). These data are supported by the protein expression data shown in Fig. 3 demonstrating that the expressed enzyme is functional, and that the catalytically dead mutant S127A (nor EGFP) has no significant effect on esterase activity.

### SIAE expression leads to an increase in GD3 in one of two clones

**Figure 5:**
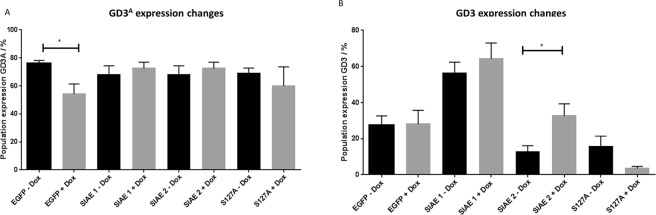
GD3 expression is increased with SIAE induction in RES256 cells. (**A**) Using flow cytometry expression of GD3^A^ was found to be significantly reduced in EGFP expressing controls (p < 0.0180). No difference was seen in SIAE clones 1, 2 or S127A. **(B)** GD3 expression was significantly increased in SIAE clone 2 (p = 0.0171). No significant changes are seen in SIAE 1, S127A or EGFP expressing cells. SIAE expression was induced for 48 hours with doxycycline. Samples were analysed using a multi-parameter fluorescence-activated cell sorting (FACS) Calibur flow cytometer and CellQuest Pro software. Data analysed by one-way analysis of variance followed by Tukey’s multiple comparisons post-hoc test using Graph Pad (^*^p < 0.05; ^**^p < 0.01) n = 3 ± SEM.

We then investigated the effects of SIAE induction on GD3^A^ and GD3 levels. Surprisingly GD3^A^ expression did not change in RES256 SIAE clones despite SIAE expression being induced by doxycycline (Fig. 5A). There was however a significant decrease in GD3^A^ in the EGFP vector control clone (Fig. 5A), the reason for which is currently unknown. Expression in the catalytically dead mutant remained unchanged. Despite no significant reduction in GD3^A^ levels, GD3 expression was found to be significantly increased in SIAE clone 2, following addition of doxycycline (p = 0.0171; Fig. 5B). An increase in GD3 expression in SIAE expressing clone 1 failed to reach significance (Fig. 5B; p > 0.05 SIAE1). Despite the significant reduction of GD3^A^ in EGFP clones, a subsequent increase in GD3 was not observed in these cells, nor was a significant change in GD3 expression in S127A cells (Fig. 5B).

### SIAE induced expression leads to collapse of the mitochondrial membrane potential

**Figure 6:**
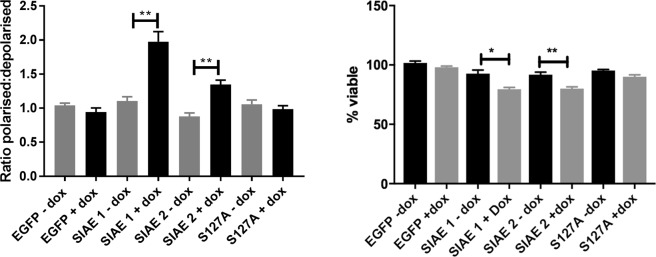
SIAE induction leads to mitochondrial membrane depolarisation in RES256 cells. (**A**) Status of the mitochondrial membrane potential was determined using the JC-1 assay after 48 hours of induction with doxycycline. Membrane polarisation was determined between cells cultured in the presence and absence of doxycycline as a ratio between red fluorescent signal (polarised) and green fluorescent signal (depolarised). Expression of wild-type SIAE significantly depolarises the mitochondrial membrane potential in clone 1 (p = 0.0058) and clone 2 (p = 0.0045). Depolarisation did not occur in EGFP or S127A expressing clones (p > 0.05). (**B**) Trypan blue exclusion determined that SIAE wild-type clones have significantly reduced viability compared to non-induced controls (SIAE 1 p = 0.0173; SIAE 2 p = 0.0097). **(A**) Data analysed by one-way analysis of variance (ANOVA) and Tukey’s post hoc test. **(B)** Data analysed using two-tailed t-test. Both analyses were carried out using GraphPad prism 6 software ^*^p < 0.05; ^**^p < 0.01), n = 3 ± SEM

We next explored the effects of SIAE induced expression on mitochondrial membrane potential using the JC-1 assay. Despite the differences seen in GD3 expression in the two SIAE clones (Fig. 5), there was a significant increase in the depolarisation of the mitochondrial membrane potential when SIAE expression was induced with doxycycline (p = 0.0058 clone 1 and p = 0.0045 clone 2; Fig. 6A). In EGFP and S127A clones, induction of expression with doxycycline did not significantly affect the mitochondrial membrane potential (p > 0.05; Fig. 6A). These data suggest that SIAE induction may result in changes in the GD3 to GD3^A^ ratio, and the increase in GD3 may result in a pro-apoptotic phenotype by mitochondrial membrane depolarisation. We then investigated if SIAE induction led to a commitment to cell death using the trypan blue exclusion method. In order to normalise for any differences in proliferation between clones, viable cells were counted just prior to doxycycline addition (0 h). After 96 hours the percentage of viable cells for each condition revealed that wild-type SIAE expression alone led to a significant reduction in cell viability (SIAE 1 p = 0.0173; SIAE 2 p = 0.0097) (Fig. 6B).

### SIAE induction and sensitisation of cells to etoposide

**Figure 7:**
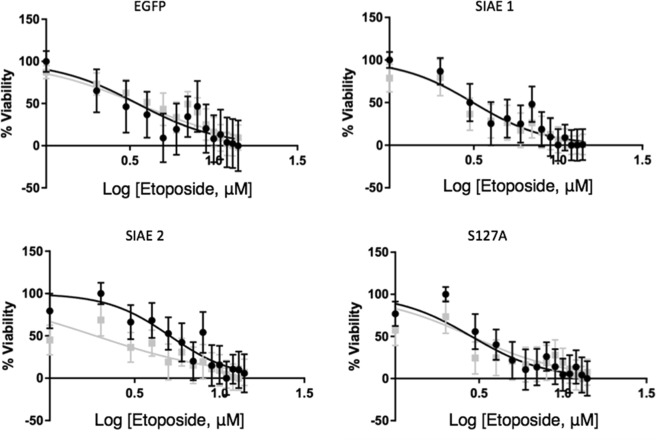
Response to etoposide is altered in SIAE over-expressing cells. RES256 cells were exposed to etoposide for 72 hours after 48 hours of induction with SIAE. No significant differences in response to drug were seen with induction of expression (induced expression is shown in grey) in EGFP, SIAE 1 or S127A (p > 0.05), however the IC_50_ was significantly reduced from 5.1 μM (±1.3) to 1.9 μM (±0.9) in SIAE 2 (p = 0.0034). Data analysed using GraphPad Prism 6 software using sigmoidal dose-response comparison analysis. Data presented as mean ± SEM n = 3.

We next sought to determine if SIAE could sensitise cells to etoposide, a chemotherapeutic with a shared mechanism of action to GD3. Etoposide is a commonly used chemotherapy for MB and is known to act in part by collapsing the mitochondrial membrane potential^36^. We therefore treated cells with etoposide to determine if induction of SIAE could sensitise cells. When clones were treated with various concentrations of etoposide for 72 hours there were no significant differences when SIAE expression was induced in any of the clones (p > 0.05; Fig. 7), except for SIAE clone 2, (p = 0.0034; Fig. 7). In these cells the IC_50_ was significantly reduced from 5.1 µM without induction to 1.9 µM with induction of SIAE expression (p = 0.0034; Fig. 7).

## Discussion

Treatment protocols for MB often leave patients with significant treatment related sequelae, including neurocognitive deficits, which severely impact quality of life^9^. There is a great need for novel therapeutic approaches, reducing the levels of toxicity arising from current treatments, and new therapeutic strategies aimed at specific MB subgroups. GD3, an oncofetal ganglioside, plays a critical role in development^13,14,22,37^. GD3 is expressed highly in several cancers where it is commonly acetylated to GD3A^24,26,28,38–41^. We determined that this pathway may have clinical relevance due to its dysregulation in MB compared to non-neoplastic cerebellum. It was found that expression the expression of CASD1 and SIAE are inversely correlated in MB subgroups groups 3 and 4, suggesting that the balance of GD3 to GD3^A^ may play a role. As CASD1 is not the only enzyme shown to be capable of acetylating GD3^29^ our efforts focused on deacetylation of GD3^A^ by means of inducing expression of SIAE induction. The catalytic site of SIAE has been described previously, allowing us carry out a functional study using this enzyme and a catalytically dead mutant SIAE S127A^42,43^.

SIAE in its active form is a glycoprotein with a molecular weight of 62 kDa^42^. When SIAE and S127A were expressed in the RES256 cell line, the expression appeared to be somewhat different, even when considering that total protein loading was less for S127A. The active glycosylated form of SIAE was expressed by both SIAE clones. An additional immuno-reactive protein was seen at 32 kDa, which was intense for the SIAE clones and less intense for S127A. The identity of 32 kDa band is unknown. GD3^A^ was significantly reduced upon doxycycline treatment in the EGFP clone, but none of the other clones demonstrated any significant differences. We also note that there was no decrease in GD3^A^ expression with SIAE expression despite SIAE clone 2 having increased GD3. One possible explanation for this has been shown in studies using endogenous addition of GD3, in which authors demonstrated that GD3 can induce its own acetylation machinery^44^. We theorise that this could be replenishing the pool of GD3^A^ and may help to explain our results.

GD3 expression has been shown to result in mitochondrial membrane depolarisation through trafficking to the mitochondrial membranes and the opening of the mitochondrial membrane permeability pore^19–21^. Induction of SIAE induction resulted in the collapse of the mitochondrial membrane potential suggesting that the increase in GD3 by SIAE was sufficient for this phenotype. We also demonstrate a commitment to apoptosis through trypan blue staining, suggestive of mitochondrial membrane potential collapse being a potential mechanism.

GD3^A^ expression has previously been shown to correlate with chemo-resistant disease^41^. The treatment regime for MB includes lomustine, cyclophosphamide, vincristine, cisplatin, and etoposide, among others^45^. As a mechanism of action of etoposide has been described in isolated mitochondria from Jurkat cells^36^ where the mitochondrial membrane potential was shown to collapse with high concentrations of etoposide (50 µM), we utilised this drug. Using clinically achievable doses of etoposide^46^ SIAE clone 2 was the only clone to demonstrate a reduced IC50 of etoposide, which may be as a result in a significant increase in GD3 expression. This data indicates a possible role for GD3 in chemo-sensitivity to etoposide, however further work is required to validate this as a mechanism of action.

## Conclusion

The GD3 acetylation pathway is re-expressed in some malignancies including MB. It has been shown in various cancers to influence cell viability, invasion and chemo-resistance. Here we have conducted a ‘proof-of-principle’ study demonstrating that the GD3 acetylation pathway could be targeted as a potential therapeutic strategy for paediatric MB as SIAE expression leads to changes in the GD3 to GD3^A^ ratio which may lead to mitochondria-mediated apoptosis and etoposide sensitivity. Additional studies are required to further demonstrate the mechanism of action of SIAE in chemo-resistance, invasion and its potential role in molecular subgroups.

## Materials and Methods

### Bioinformatics

All bioinformatics analysis was carried out using publically available data sets from the R2 database (r2.amc.nl). Specific datasets used for each figure are described in the corresponding figure legends. The datasets used were Roth (GSE3526)^34^; Pfister (GSE49243)^47^, Gilbertson (GSE37418)^48,49^; Northcott (GSE21140)^35^; MAGIC (GSE37382)^50^ and Cavalli (GSE85217)^51^.

### Cell culture

The paediatric MB group 4 cell line CHLA-01-MED and the metastatic melanoma cell line MeWo were purchased from the American Type Culture Collection (ATCC). The cell lines RES256 and UW402 were obtained through material transfer agreement with Dr. John Sibler, Seattle. Cell lines were DNA fingerprinted using an in-house method^52^ and compared to published genotypes where available to validate cell line identity. Cell lines were confirmed as mycoplasma free using the MycoAlert detection kit (Lonza). RES256 and UW402 cells were cultured in Dulbecco’s modified Eagle’s media (DMEM) (Gibco) supplemented with 10% foetal bovine serum (FBS) (Sigma). CHLA-01-MED was cultured according to distributor’s recommendation as a suspension culture in DMEM f-12 (ATCC) supplemented with 2% v/v B27 supplement (Invitrogen) and 20 ng/mL FGF-2 and EGF (Miltenyi Biotech). MeWo was cultured according to distributor’s recommendation in minimum essential media (MEM; ATCC) supplemented with 10% FCS. Cells transfected with pCMV-TetOn3G were selected with 1 mg/mL G-418 (Source Bioscience), cells also transfected with pTRE3G-IRES and linear puromycin marker constructs were selected with 0.5 ng/mL puromycin (Life technologies). To induce expression cells were cultured in the presence of 1 µg/mL doxycycline (Clontech) for 48 hours in a humidified 5% CO_2_ 37 °C incubator. Transfected clones were cultured in DMEM supplemented with 10% tetracycline-free FCS (Clontech).

### Cloning

pCMV-TetOn3G and pTRE3G-IRES constructs were purchased from Clontech. EGFP, SIAE and SIAE S127A genes were synthesised by Eurofins Genomics. Each gene was amplified using CloneAmp PCR premix (Clontech) and the following primers which contain 15 bp overhang regions complementary to the linearized plasmid and restriction sites SIAE-F CCCTCGTAAAGTCGACATGGTCGCGCCGGGGCTTGTACTC; SIAE-R GGAGAGGGGCCGGCCGTCATTTAGCAACATTGCTCTGATG; EGFP-F GCCGGATATCACGCGTATGGTGAGCAAGGGCGAGGAGCTG; EGFP-R CAGTTACATTGGATCCTTACTTGTACAGCTCGTCCATGCC.

PCR cycling conditions were as follows; melting temperature 98 °C for 10 seconds, annealing temperature 55 °C for 15 seconds, extension temperature 72 °C for 8 seconds for 35 cycles. The PCR products were spin column purified using the Nucleospin PCR purification kit according to manufacturer’s instructions (Clontech). The pTRE3G-IRES constructs were then linearized using restriction digests with SalI and EagI for EGFP cloning, or MluI/BamHI for SIAE or S127A cloning (NEB) and then spin column purified as previous. HD EcoDry InFusion cloning (Clontech) was then used to insert amplified genes into linearized constructs by incubation in a BioRad Thermal cycler for 15 minutes at 37 °C, followed by 15 minutes at 50 °C. The resulting DNA was transformed into Stellar competent *E. coli* (Clontech) according to manufacturer’s instructions. Plasmid DNA obtained was isolated using a PureYield plasmid miniprep kit (Promega) and sequenced by Eurofins genomics to ensure sequence integration and accuracy.

### Generation of double stable transfectants

Cell lines were transfected Xfect transfection reagent (Clontech) with pCMV-TetOn3G constructs (Clontech) and selected with 1 mg/mL G-418 (Biochrom). Resulting colonies were expanded and screened for leaky expression and inducibility by transient transfection with tet-responsive luciferase constructs pTRE3G-Luc (Clontech) in the presence and absence of 1 μg/mL doxycycline (Clontech) for 48 hours to induce gene expression. The samples were then assayed using the Dual Luciferase assay kit (Promega) according to manufacturer’s instructions. The selected clone was then co-transfected with pTRE3G-IRES containing cloned genes of interest and with a linear puromycin marker. Clones were selected with 0.5 μg/mL puromycin (Fisher) and selected using fluorescence microscopy (selection using EGFP reporter). Constructs were tet-responsive- pTRE3G-IRES-EGFP (empty vector control); pTRE3G-IRES-EGFP-SIAE (which generated two clones) or pTRE3G-IRES-EGFP-SIAE-S127A (active site mutant).

### SIAE antibody production

An immunogenic candidate SIAE peptide was designed using NHLBI-AbDesigner software. Alta Bioscience Ltd produced the selected 17 amino acid peptide sequence (SSDLSKKSSDDGFPQIR) which had optimal immunogenicity and minimal cross-reactivity. The SIAE peptide was used to produce antibodies in sheep by National Health Service (NHS) Scotland. Sheep were bled and the antibody was purified for use in this project.

### Protein isolation and western blot analysis

Protein was extracted 48 hours after doxycycline treatment using M-PER lysis buffer (Thermo) supplemented with 1% phosphatase and protease inhibitors (Thermo). Protein concentrations were determined using BCA assay (Thermo) on an Optima plate reader (BMG Labtech). Proteins were then resolved using 10% Tris-gycine gels (Thermo). After separation, protein (50 µg) was transferred to a PVDF-ImmunoBlot membrane (BioRad) and blocked in 5% non-fat milk for 1 hour at room temperature on a rocking platform. The blots were then incubated with primary antibody overnight at 4°C on a rocking platform. Antibodies were applied as follows: sheep anti-human SIAE (Custom, see antibody production) 3 ug/mL and mouse anti-human β-actin (Sigma). Subsequently, the blots were washed five times in Tris-buffered saline containing 0.05% Tween-20 before and after 1 hour of incubation at room temperature with Licor 800CW infared secondary antibodies. Blots were performed in dupicate. β-actin was used to control for protein loading. Blots were imaged by Licor Odyssey Clx technology using Image Studio 5 software.

### Flow cytometry

Expression of GD3 and GD3^A^ was determined by flow cytometry. Cells were cultured in 1.5 mL media and treated as per experiment, cells were seeded at 250,000 cells per well. Cells were harvested and centrifuged at 300 rcf for 5 minutes at 4 °C in an Eppendorf 5145R centrifuge. Cells were then resuspended in 1 mL ice-cold phosphate buffered saline (PBS; Sigma) supplemented with 5% normal goat serum (NGS; Biosera) and centrifuged using the same conditions as previously described, twice (wash step). Cells were permeabilized using 250 µL Cytofix/Cytoperm solution (BD Biosciences) at 4 °C for 20 minutes before incubating with primary antibody for 30 minutes at 4 °C (20 µg/mL mouse anti-human GD3 clone MB3.6 (Millipore) or 10 µg/mL mouse anti-human 9-*O*-acetyl GD3 (Thermo)) in the presence of 5% goat serum and 0.1% saponin (Sigma). The wash step was repeated. Next cells were incubated with 4 μg/mL goat anti-mouse secondary antibody, AlexaFluor 488 (Invitrogen) in the presence of 0.1% saponin for 15 minutes at 4 °C. The wash step was repeated. Cells were then resuspsended in 300 µL cold PBS supplemented with 2% NGS and 0.01% sodium azide (Sigma), and filtered using 20 µm nylon mesh filter (Millipore). Expression data were collected using a 4-color multiparameter fluorescence-activated cell sorting (FACS) Calibur flow cytometer (BD Biosciences), and acquisition and analysis of data were carried out using CellQuest Pro Software.

### JC-1 Assay

Cells were seeded at 250,000 per well, in the presence and absence of doxycycline. After 48 hours, cells were harvested and centrifuged at 300 rcf for 5 minutes. Each sample, re-suspended cell in 1 ml of PBS (Sigma) and 12.5 µl of JC-1 (Chemometec) at a final concentration of 2.5 µg/ml and incubated for 10 minutes at 37 °C. The stained cells were centrifuged at 400 rcf for 5 minutes at room temperature and remove the supernatant completely without disturbing the cell pellet. The cell pellet was re-suspended in 1 ml PBS by pipetting, centrifuge at 400 rcf for 5 minutes room temperature and the supernatant removed completely without disturbing the cell pellet, this is repeated a second time. Finally, the cell pellet is re-suspended in 0.25 ml DAPI (1 µg/ml) and fluorescence intensity measured immediately in a black 96 well plate, Green (Excitation/Emission 485 nm/520 nm) and Red (Excitation/Emission 544 nm/590 nm) using BMG OPTIMA PolarStar. In healthy cells, the negative charge established by the intact mitochondrial membrane potential causes an accumulation of JC-1 in the mitochondrial matrix. At high concentrations JC-1 forms aggregates and become red fluorescent. In apoptotic cells the mitochondrial potential collapses and JC-1 localises to the cytosol in its monomeric green fluorescent form. The ratio of aggregates (red) to monomers (green) was calculated.

### Trypan blue exclusion assay

RES256 cells were plated at 250,000 cells in 6-well plates. After 24 h of adherence cells were counted using the trypan blue solution (Life Technologies) according to manufacturer’s protocol. The cells were then counted using the automated Countess II cell counter (Life Technologies). Cells were then treated with doxycycline for 96 h before cells were counted using the method previously described. Cell counts were normalised to time 0 h to normalise for proliferation differences between clones prior to doxycycline treatment and expressed as percentage viable.

### Esterase activity assay

The enzymatic activity of SIAE of transfected cells was determined using the general esterase artificial substrate 4-nitrophenyl acetate (pNPA) (Sigma), the release of the product 4-nitrophenol (pNP) (Sigma) can be measured at 405 nm, as described^28^. Cells were seeded at 2,500 cells per well in 100 μL clear media in 96 well plates, in the presence and absence of doxycycline. Firstly, a 20 mM stock of pNPA was prepared in DMSO. Media was removed and the cells were washed twice with PBS. In each well, 220ul of Phosphate buffer (50 mM, pH 7.4) was added, pNPA was added using on board injectors to a final concentration of 0.1 mM or 0.2 mM and absorbance was measured at 410 nm using BMG OPTIMA PolarStar. A standard curve was generated using pNP (0.025–0.2 mM).

### Chemotherapy experiments

Treatment of cells with etoposide was carried out by plating EGFP, SIAE and SIAE-S127A expressing clones at 2,500 cells per well in 100 μL clear media in 96 well plates. Cells were plated in the presence and absence of doxycycline. After 48 of doxycycline treatment cells were treated with Etoposide (Sigma) prepared in dimethylsulfoxide (DMSO; Sigma). Concentrations ranged from 1 μM to 14 μM in 1 μM increases. Untreated cells and vehicle controls were included. Cells were treated for 72 hours post-chemotherapy addition. Phase-contrast images were taken and MTS assays using CellTiter 96 AQ_ueous_ One Solution (Promega) were carried out at the end of the time course according to manufacturer’s instructions. Plates were incubated at 37 °C for 3 hours. The absorbance was then read at 490 nm using the BMG labtech POLARstar Optima plate reader.

### Statistics

All experiments were carried out in triplicate unless otherwise stated. All statistical analysis was carried out using GraphPad Prism 6 software, individual statistical tests are outlined as appropriate in figure legends. P-values less than 0.05 were considered significant.

## Supplementary information

Supplementary figures 1-3

## Data Availability

The datasets generated during and/or analysed during the current study are available from the corresponding author on reasonable request.
