# Supplementary material for: Human Sialic acid O-acetyl esterase (SIAE) – mediated changes in sensitivity to etoposide in a medulloblastoma cell line
Source: Sci Rep. 2019 Jun 13;9:8609. doi: 10.1038/s41598-019-44950-5 (PMC6565703; doi:10.1038/s41598-019-44950-5)
Supplement: Supplementary file 1 — Supplementary figures 1-3 [file 41598_2019_44950_MOESM1_ESM.pdf]

Supplementary figures

Human Sialic acid *O*-acetyl esterase (SIAE) – mediated changes in sensitivity to etoposide in a medulloblastoma cell line

Mather RL, Loveson KF, Fillmore HL

Supplementary figure 1

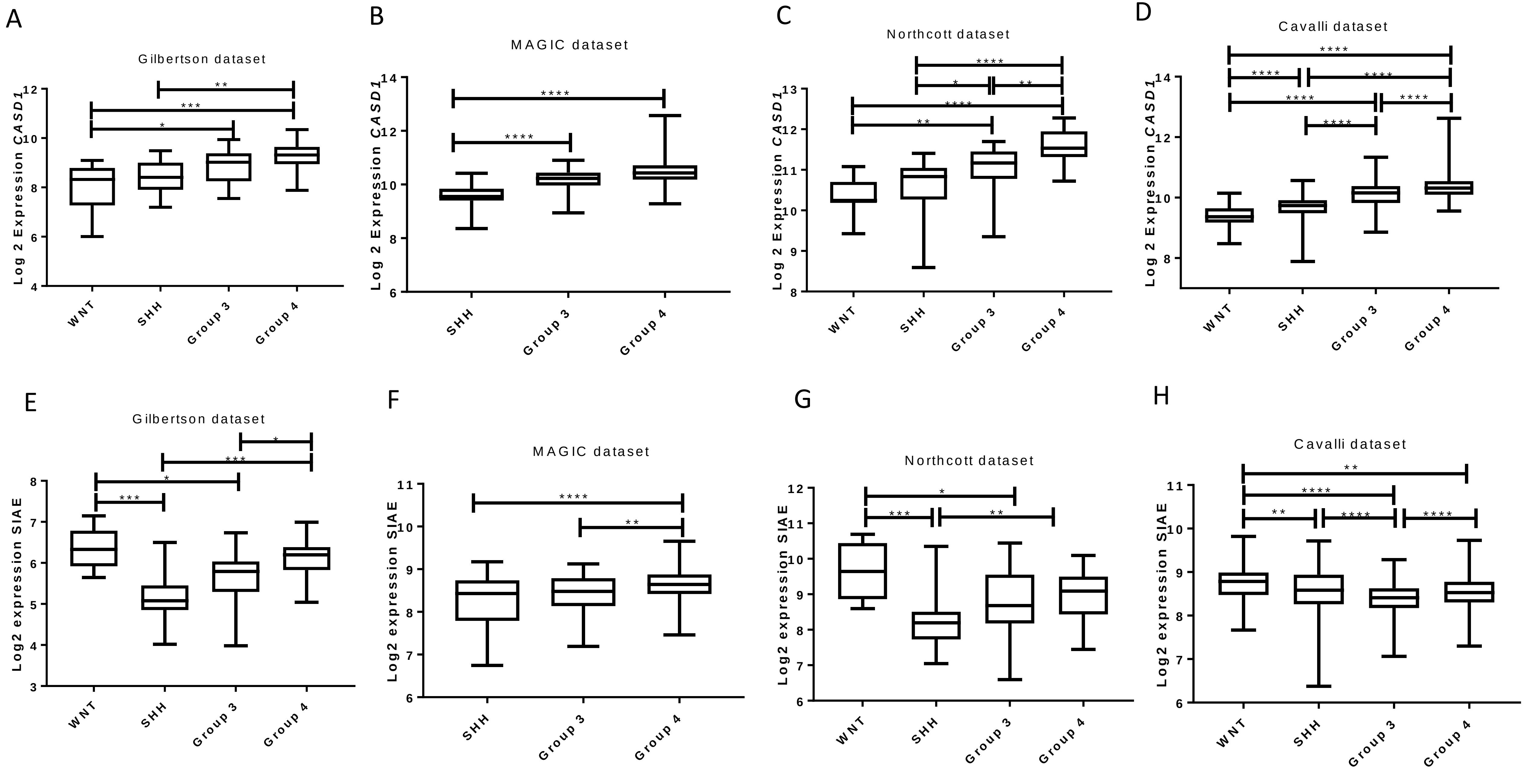

Supplementary Figure 1: The GD3 pathway is consistently dysregulated across datasets. (A) Data from the Gilbertson MB dataset (gse37418) demonstrates that expression of CASD1 is significantly higher in group 4 compared to WNT (p=0.0001), and SHH (p=0.0032). Expression of CASD1 is also significantly higher in group 3 compared to WNT (p=0.0235). N=76 **(B)** Data from the MAGIC consortium dataset (gse37382) demonstrates higher expression of CASD1 in groups 3 and 4 compared to SHH (p<0.0001) n=285.**(C)** Data from the Northcott core exon dataset (gse21140) demonstrates highest expression in group 4 (WNT p<0.0001; SHH p=<0.0001; group 3 p=0.0011); as well higher expression in group 3 compared to WNT (p=0.0025) and SHH (p=0.0106) n=103 **(D)** Data from the Cavalli dataset (gse85217) demonstrates higher expression of CASD1 in groups 3 and 4 compared to SHH (p<0.0001) n=763. **(E)** Expression of SIAE is highest in the WNT subgroup compared to SHH (p=0.0003) and group 3 (p=0.0147). SIAE is also significantly lower in SHH tumours compared to group 4 (p=0.0001), and is significantly lower in group 3 compared to group 4 (p=0.0127) n=76. **(F)** Expression of SIAE is lower in SHH tumours compared to group 4 (p<0.0001), as well as SIAE expression being lower in group 3 compared to group 4 (p=0.0075) n=285. **(G)** Expression of SIAE is highest in WNT tumours compared to SHH (p=0.0001) and group 3 (p=0.0359), as well as being lower in SHH compared to group 4 (p=0.0019) n=103. **(H)** Expression of SIAE is highest in WNT tumours compared to SHH (p=0.0058), group 3 (p<0.0001), and group 4 (p=0.0016), as well as being lower in SHH compared to group 4 (p<0.0001), and lower in group 3 than group 4 (p<0.0001) n=763. Data analysed by one-way analysis of variance (ANOVA) followed by Tukey's multiple comparisons post hoc test using graph pad prism 6 software. (* p<0.05; ** p<0.01; **** p<0.0001).

Supplementary figure 2

-Doxycycline +Doxycycline

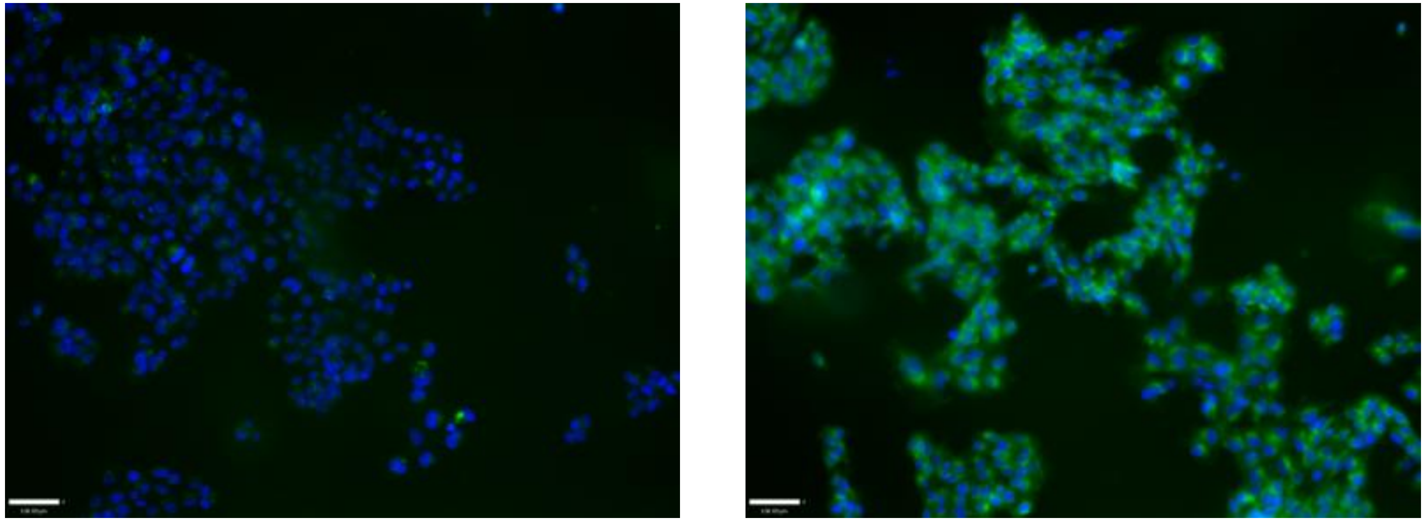

Supplementary figure 2: EGFP expression is induced in SIAE clones. A representative epifluorescence image of the indirect screening of RES256 cells transfected with pTRE3G-IRES-EGFP-SIAE after 48 h induction with doxycycline. SIAE expression was later confirmed in all EGFP-positive clones by western blot (figure 3). Nuclei counterstained with Hoechst blue. Images were taken at 10 X magnification using Zeiss Axioimager ZI epfluorescence microscope equipped with a Hamamatsu digital camera and Volocity imaging software.

A

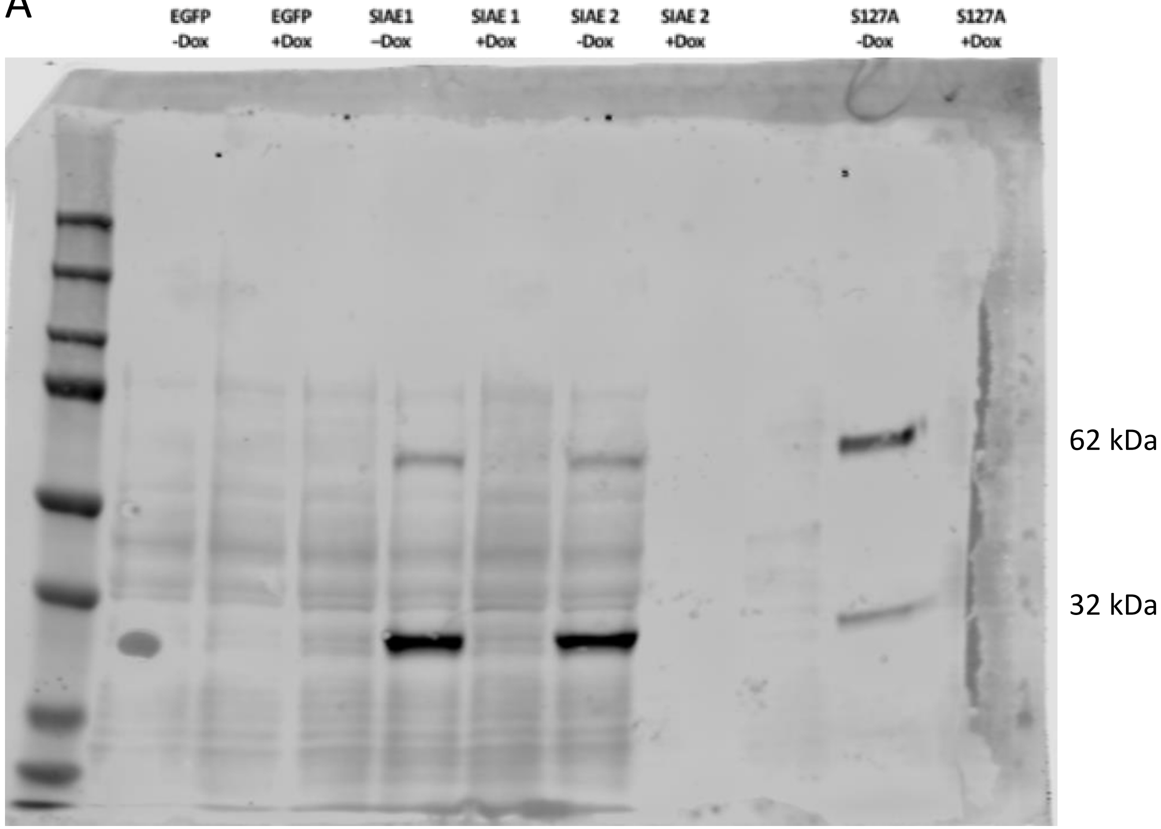

B

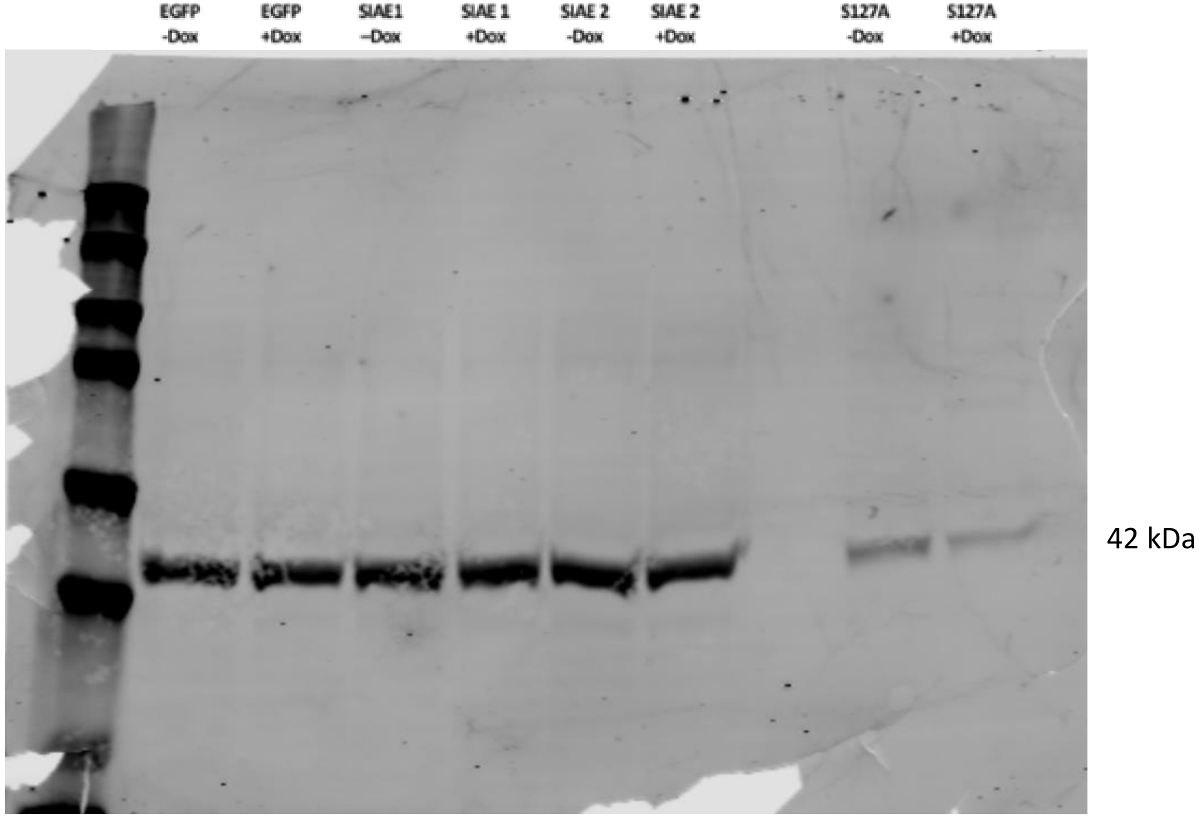

Supplementary figure 3: Full image of western blot shown in figure 3. RES256 cells were cultured in the presence (+) and absence (-) of doxycycline (dox) for 48 hours. **(A)** SIAE was expressed by two wild-type clones, SIAE 1 and SIAE 2, and by the catalytic mutant, S127A only when the cells were cultured with doxycycline. **(B)** Protein loading between samples from each clone are confirmed with beta actin. A further immuno-reactive band of approximately 32 kDa for SIAE was seen. The identity of this band is unknown. Less protein was loaded in the S127A samples due to protein concentration of the lysate. Image acquired using the Licor Odyssey Clx equipped with ImageStudio 5 software. Representative image of n=2.
